# Dexmedetomidine Added to Sufentanil Patient-Controlled Intravenous Analgesia Relieves the Postoperative Pain after Cesarean Delivery: A Prospective Randomized Controlled Multicenter Study

**DOI:** 10.1038/s41598-018-27619-3

**Published:** 2018-07-02

**Authors:** Yuyan Nie, Weifeng Tu, Xiaofeng Shen, Weifeng Yu, Yonghao Yu, Xingrong Song, Shiduan Wang, Ailin Luo, Minghui Cao, Xinmin Wu, Shaoqiang Huang

**Affiliations:** 1 Department of Anaesthesiology, the Obstetrics and Gynecology Hospital of Fudan University, Shanghai, 200090 China; 2 Department of Anaesthesiology, General Hospital of Guangzhou Military Command of PLA, Guangzhou, 510010 China; 3 Department of Anaesthesiology, Nanjing Maternity and Child Care Hospital, Nanjing, 210000 China; 4 Department of Anaesthesiology, Renji Hospital Shanghai Jiaotong University School of Medicine, Shanghai, 200127 China; 5 Department of Anaesthesiology, Tianjin Medical University General Hospital, Tianjin, 300052 China; 6 Department of Anaesthesiology, Guangzhou Women And Children’s Medical Center, Guangzhou, 510623 China; 7 Department of Anaesthesiology, The Affiliated Hospital of Qingdao University, Qingdao, 266071 China; 8 Department of Anaesthesiology, Tongji Medical College Huazhong University of Science & Technology, Wuhan, 430030 China; 9 Department of Anaesthesiology, Sun Yat-Sen Memorial Hospital of Sun Yat-Sen University, Guangzhou, 510120 China; 10 Department of Anaesthesiology, Peking University First Hospital, Peking, 100034 China

## Abstract

This study evaluated the efficacy and safety of dexmedetomidine in intravenous patient-controlled analgesia (PCA) after cesarean delivery. This multicenter study enrolled 208 subjects who were scheduled for selective cesarean delivery from 9 research centers. Patients received 0.5 ug/kg dexmedetomidine (study group) or normal saline (control group) after delivery and an intravenous PCA pump after surgery (100 μg sufentanil +300 μg dexmedetomidine for the study group, 100 μg sufentanil for the control group, background infusion: 1 ml/h, bolus dose: 2 ml and lock time: 8 min). The sufentanil consumption, pain scores, rescue analgesia, sedation scores, analgesic satisfaction, the incidence of postoperative nausea and vomiting (PONV) and the first passage of flatus were recorded within 24 h after surgery. The sufentanil consumption in the study group was significantly lower than that in the control group (p = 0.004). Compared with the control group, the study group had lower pain scores (p < 0.01), higher analgesic satisfaction degree [p < 0.001, odd ratio 4.28 and 95% CI (2.46, 7.46)], less requirement of rescue analgesia (p = 0.003), lower incidence of PONV (p = 0.005 and p < 0.001, respectively), and shorter time to first passage of flatus (p = 0.007). Dexmedetomidine added to sufentanil intravenous PCA significantly enhanced the analgesic effects, improved analgesic satisfaction, and had the potential benefits of reducing PONV and the recovery of intestinal functions after cesarean section.

## Introduction

Multimodal analgesia using strong opioids combined with non-opioid analgesics is the most common technique for postoperative pain control after cesarean delivery. This multimodal analgesic method aims to improve analgesic efficacy, decrease postoperative opioid requirements, and subsequently, opioid-related adverse effects. Additionally, it has been claimed that this opioid-sparing analgesic method may contribute to reduce the occurrence of postoperative hyperalgesia^1^. Sufentanil, a selective µ-receptor agonist, is commonly used for patient-controlled analgesia (PCA) because of its rapid peak, powerful analgesic activity and short half-life^2,3^.

Dexmedetomidine is a highly selective α2 receptor agonist with obvious analgesic, sedative, and anti-sympathetic functions^4,5^. Additionally, dexmedetomidine can increase the frequency and amplitude of uterine smooth muscle contractions^6^. Though some isolated reports revealed that dexmedetomidine has been used in the care of obstetric patients including general anesthesia in cesarean delivery^7^, labor analgesia^8,9^, and postoperative analgesia after cesarean section^10^, the systemic use of dexmedetomidine has not been well studied in the postpartum population. Our previous study found that a loading dose dexmedetomidine after delivery supplemented with postoperative sufentanil combined with dexmedetomidine for intravenous PCA enhanced the analgesic effects and improved the analgesic satisfaction of parturients^10^. Dexmedetomidine as a α2 receptor agonist could induce bradycardia and excessive sedation. In addition to the analgesic effects, the safety is particularly important for postoperative analgesia. Moreover, whether the addition of dexmedetomidine in the analgesic regimen would provide extra benefits, such as uterine contraction pain relief and/or contribution to the recovery of intestinal functions, is unknown. Considering the special population pharmacokinetics of dexmedetomidine in parturients, more powerful evidence on the safety of dexmedetomidine in obstetric patients was also needed. Therefore, potential applications for dexmedetomidine in this population certainly deserve further study. This study was conducted in multi-centers with more subjects enrolled to test the hypothesis that dexmedetomidine added to sufentanil PCA may improve postoperative analgesia and be beneficial in terms of postoperative nausea and vomiting (PONV) and the intestinal function after cesarean section.

## Results

**Figure 1:**
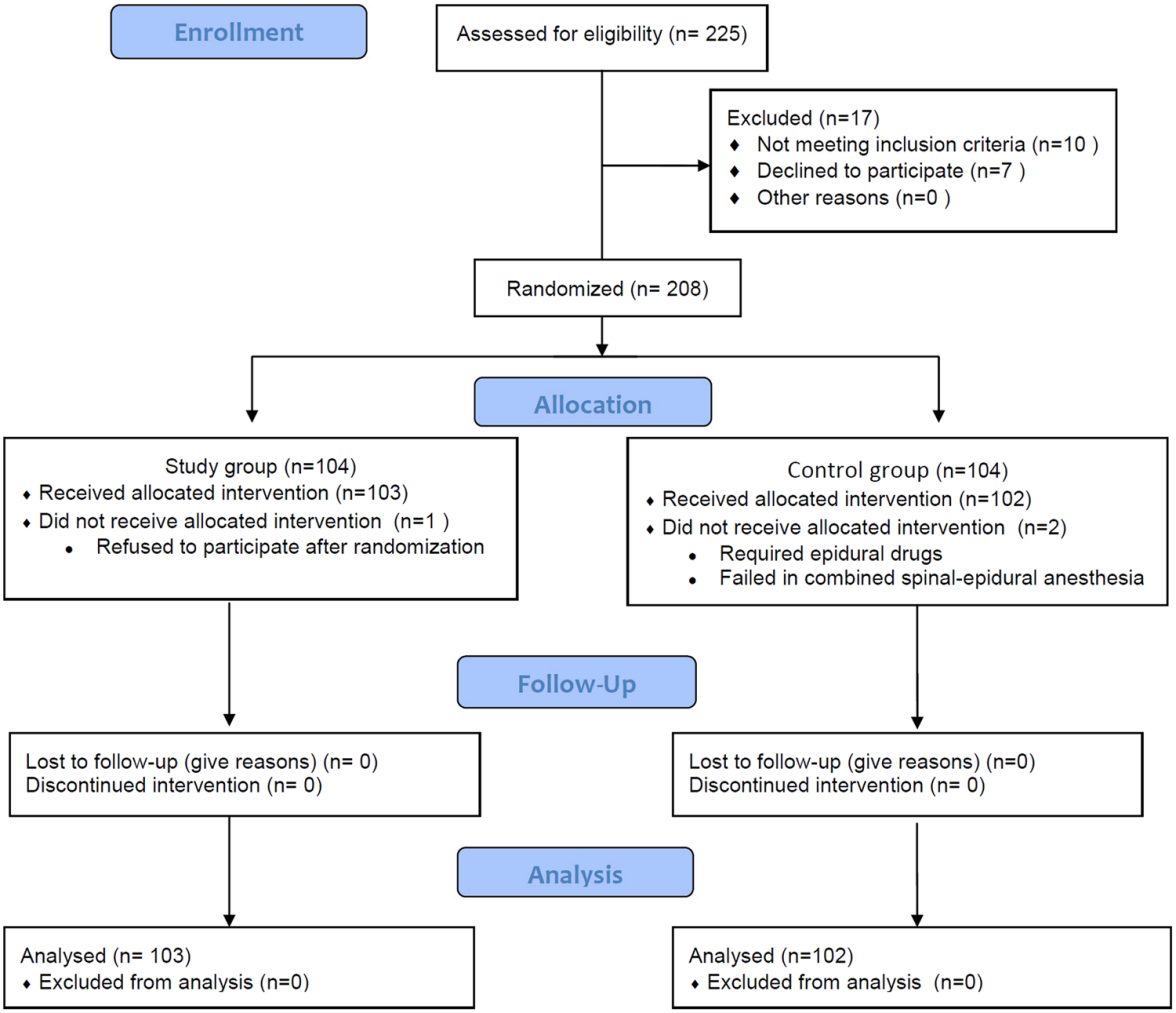
CONSORT recruitment flow diagram.

From June 2015 to January 2016 a total of 225 parturients were assessed for enrollment in 9 research centers. Two hundred and eight parturients were randomly assigned. One case in the study group withdrew from this study after randomization. In the control group, 2 cases were excluded, 1 case required epidural drugs to achieve surgical anesthesia effects, and 1 case experienced general anesthesia because of failure in combined spinal-epidural anesthesia (CSEA). A total of 205 parturients finally completed this study and were included in the statistical analysis, including 102 cases in the control group and 103 cases in the study group (see Fig. 1).

Clinically important differences in baseline characteristics including age, height, body weight, body mass index (BMI), gestational age, spinal bupivacaine /ropivacaine and surgical time were not apparent in the two groups (Table 1).

**Table 1:** Maternal Demographics. Values are mean (SD) or number (proportion). BMI, Body Mass Index.

	Control Group (n = 102)	Study Group (n = 103)	p
Age (yr)	30.9 (4.3)	30.2 (4.4)	0.19
Height (cm)	161.0 (4.3)	161.3 (4.8)	0.64
Weight (kg)	69.4 (7.1)	69.7 (8.8)	0.80
BMI (kg/m^2^)	26.8 (2.4)	26.7 (2.6)	0.90
Gestational age (wk)	38.7 (1.4)	38.5 (1.4)	0.32
Spinal bupivacaine (n)/ropivacaine (n)	72/30	72/31	0.48
Duration of surgery (min)	60.2 (25.4)	57.3 (23.6)	0.41

The sufentanil consumption during 24 h after surgery in the study group (44.9 ± 23.7) µg was significantly lower than that in the control group (55.0 ± 25.7) µg (p = 0.004). Comparing with the control group, the requirement of sufentanil in the study group decreased by 18.4% [(10.14 ± 3.45) µg, 95% CI (3.33, 16.95)].

**Figure 2:**
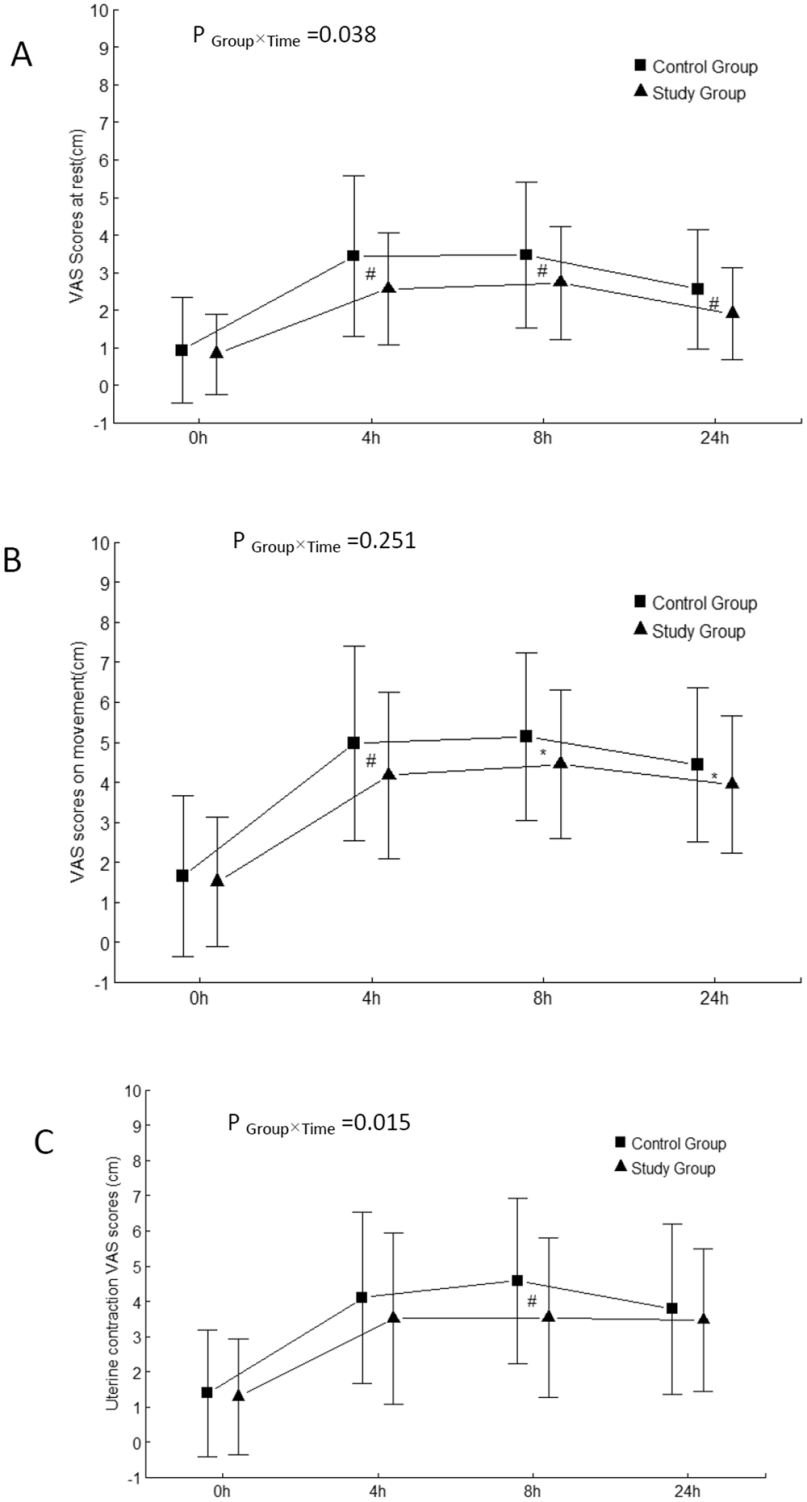
Changes in pain intensity at rest (**A**), on movement (**B**) and Uterine contraction pain (**C**) after surgery (mean and SD). VAS, the visual analog scale; P _Group×Time_, P value of the group and time interaction obtained by the linear mixed model. ^*^P < 0.05, ^#^P < 0.01.

The visual analog scale (VAS) scores at rest (overall p < 0.001), on movement (overall p = 0.007) and uterine contraction (overall p = 0.033) were significantly different between the two groups. When all time points were combined with the linear mixed models, the VAS at rest and uterine contraction during the postoperative period were still significantly lower in the study group (p = 0.038, 0.015 respectively). However, no statistically significant difference was found in VAS on movement when interaction between group and time was considered though the patients in the study group had lower VAS scores on movement at 4 h [mean difference (MD) 0.83, 95% confidence interval (CI) (0.21, 1.44), p = 0.009], 8 h [0.7 (0.16, 1.24), p = 0.011] and 24 h [0.50 (0.00, 0.99), p = 0.049] after surgery respectively (Fig. 2B). The VAS scores at rest in the study group were significantly lower than those in the control group at 4 h [MD 0.89, 95% CI (0.39, 1.39), p = 0.001], 8 h [0.75 (0.28, 1.22), p = 0.002] and 24 h [0.65 (0.27, 1.03), p = 0.001] post-surgery respectively (Fig. 2A). The uterine contraction pain score was significantly lower in the study group only at the postoperative 8 h [1.02 (0.39, 1.66), P = 0.002] (Fig. 2C).

The results for rescue analgesia and PCA pump bolus outcomes were reported in Table 2. Postoperative rescue analgesia significantly differed between the groups. Compared with the control group, the study group required less rescue analgesia within the first postoperative 24 h [p = 0.003, RR = 1.12, 95% CI (1.04, 1.22)]. Compared with the control group, the number of total bolus [4.6, 95% CI (0.74, 8.46), p = 0.02], actual PCA bolus [3.7 (0.55, 6.85), p = 0.02] and ineffective bolus [1.5 (0.07, 2.93), p = 0.04] within 24 h were lower in the study group.

**Table 2:** Postoperative rescue analgesia and PCA pump bolus outcomes (the number of bolus).

		Control Group (n = 102)	Study Group (n = 103)	P value	MD/RR	95% CI	
						Lower	Upper
Rescue analgesia	No (n)	89 (87.3%)	101 (98.1%)	0.003	1.12	1.04	1.22
	One dose (n)	11 (10.8%)	2 (1.9%)	0.009	0.18	0.04	0.79
	Four doses (n)	2 (1.9%)	0	0.229	—	—	—
PCA bolus	Total (n)	14.4 (15.7)	9.8 (12.1)^*^	0.020	4.60	0.74	8.46
	Actual (n)	11.9 (12.4)	8.2 (10.4)^*^	0.022	3.70	0.55	6.85
	Ineffective (n)	3.4 (6.4)	1.9 (3.6)^*^	0.040	1.50	0.07	2.93

Values are mean (SD) or number (proportion, %). MD, mean difference. RR, risk ratio. Postoperative rescue analgesia significantly differed between the two groups (p = 0.003). One dose rescue analgesia was 100 mg intramuscular tramadol. *Significant difference between the two groups (p < 0.05).

Patients in the study group had significantly higher levels of analgesic satisfaction than patients in the control group (p < 0.001) (Table 3). The odd ratio was 4.28 and its 95% CI was [2.46, 7.46].

**Table 3:** Satisfaction Degree for Analgesia in Two Groups.

	Control Group (n = 102)	Study Group (n = 103)
Not satisfied	22 (21.6%)	4 (3.9%)
Less satisfied	35 (34.3%)	23 (22.3%)
Satisfied;	42 (41.2%)	57 (55.3%)
Very satisfied	3 (2.9%)	19 (18.5%)

Values are number (proportion). The study group had significantly higher satisfaction degree than the control group [p < 0.001, risk ratio, 2.13 and 95% CI (1.48, 3.08)].

The Observer’s Assessment of Alertness/Sedation Scale (OAA/S) scores of the parturients postoperatively at 0, 4, 8, 24 h were not less than grade 3 and not significantly different between the groups (p > 0.05) (Table 4).

**Table 4:** Sedation Degree in Two Groups at 0, 4, 8, 24 h After Surgery.

OAA/S	Control Group (n, %)				Study Group (n, %)			
	0 h	4 h	8 h	24 h	0 h	4 h	8 h	24 h
Grade 5	94 (92.2)	96 (94.1)	93 (91.2)	95 (93.1)	90 (87.4)	88 (85.4)	91 (88.3)	93 (91.3)
Grade 4	6 (5.9)	4 (3.9)	9 (8.8)	7 (6.9)	8 (7.8)	8 (7.8)	9 (8.7)	10 (9.7)
Grade 3	2 (2.0)	2 (2.0)	0 (0)	0 (0)	5 (4.9)	7 (6.8)	3 (2.9)	0 (0)
Grade 2	0 (0)	0 (0)	0 (0)	0 (0)	0 (0)	0 (0)	0 (0)	0 (0)
Grade 1	0 (0)	0 (0)	0 (0)	0 (0)	0 (0)	0 (0)	0 (0)	0 (0)

Values are number of patients (%); OAA/S, the modified Observer’s Assessment of Alertness/Sedation Scale. There is no significant difference in sedation degree between the groups.

**Figure 3:**
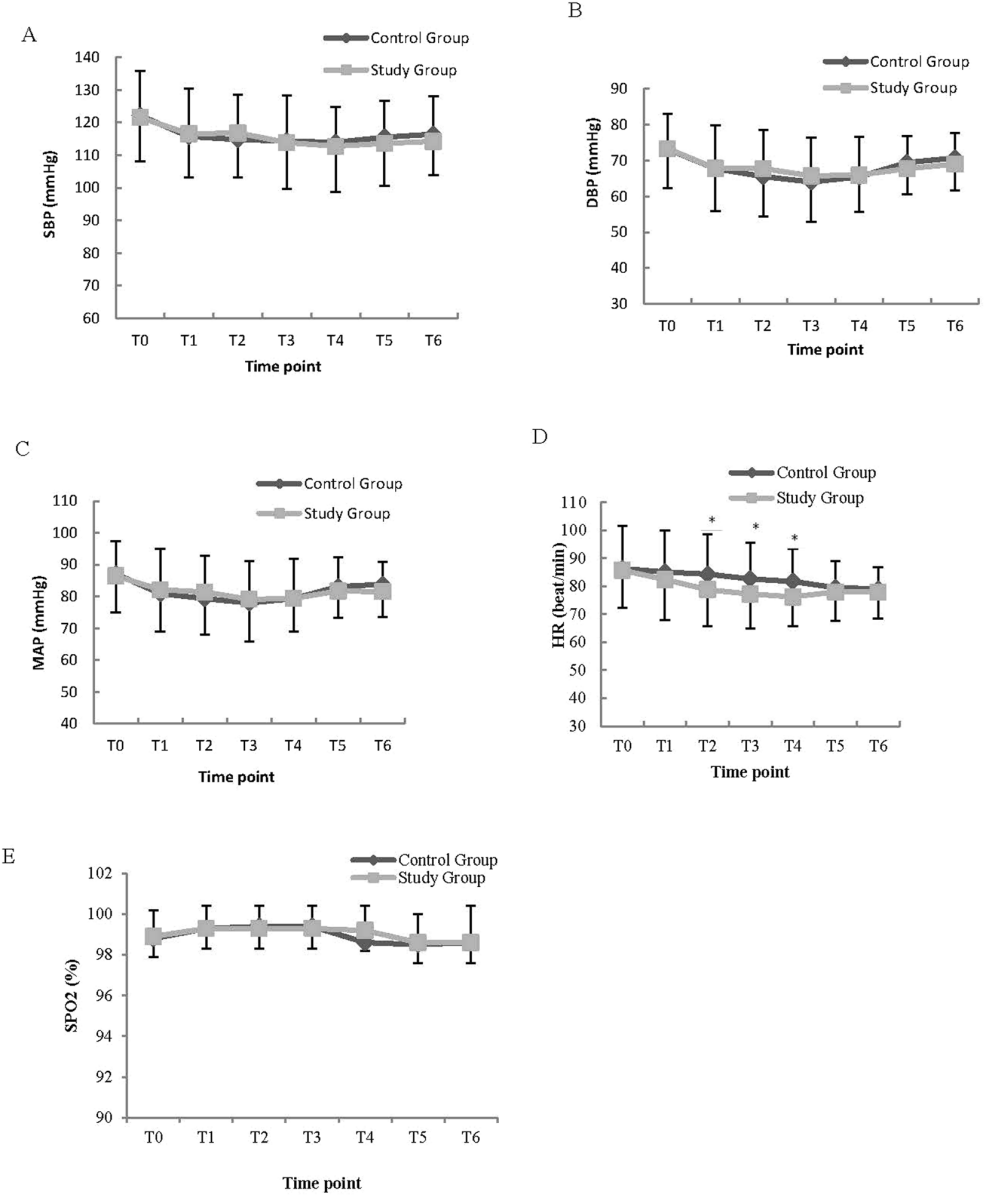
Changes in SBP (**A**), DBP (**B**), MAP (**C**), HR (**D**) and SPO2 (**E**) between two groups. SBP, systolic blood pressure; DBP, diastolic blood pressure; MAP, mean arterial pressure; HR, heart rate; SpO2, oxygen saturation. T0, Baseline; T1, T2, T3, T4, 0 min, 5 min, 10 min, 30 min after drug administration during surgery respectively; T5, T6, postoperative 8 h, 24 h respectively. *Significant difference between groups (P < 0.05).

There were no significant differences in the systolic blood pressure (SBP), diastolic blood pressure (DBP), mean arterial pressure (MAP), and oxygen saturation (SpO2) between the groups at the 7 time points (two-way ANOVA overall P, 0.67, 0.88, 0.61, 0.41 respectively) (Fig. 3A–C,E). The heart rates (HRs) were significantly different between the two groups (two-way ANOVA overall P = 0.01). HRs in the study group at 5 min (p = 0.003), 10 min (p = 0.001), and 30 min (p < 0.001) after the loading dose of dexmedetomidine were significantly lower than those in the control group during surgery. However, no significant differences were detected between the groups in heart rate (HR) at 8 h (p = 0.29) and 24 h (p = 0.37) after surgery (Fig. 3D).

The incidence of nausea [p = 0.005, RR = 0.48, 95%CI (0.28, 0.82)] and vomiting [p < 0.001, RR = 0.25, 95%CI (0.11, 0.54)] in the study group was significantly lower than that in the control group. The time to first passage of flatus after surgery in the control group was significantly longer compared to that in the study group (p = 0.008). The amount of lochia discharged within the first 3 h after surgery and the onset of lactation was not significantly different between the groups (p = 0.432) (Table 5).

**Table 5:** Other Maternal outcome after operation and Summary of Adverse Events.

	Control Group (n = 102)	Study Group (n = 103))	P value
First passage of flatus (h)	26.0 (11.9)	21.8 (10.4)^*^	0.008
Lochia volume (ml)	65.3 (84.7)	74.5 (82.6)	0.432
Initiation of lactation (h)	34.2 (23.9)	32.6 (24.9)	0.639
Nausea (n, %)	33, (32.3%)	16, (15.6%)^*^	0.005
Vomiting (n, %)	28, (27.1%)	7, (6.54%)^*^	<0.001
Hypertention (n)	2	0	—
Hypotention (n)	0	0	—
Tachycardia (n)	1	1	—
Bradycardia (n)	0	1	—
Respiratory depression	0	0	—
Hypoxemia	0	0	—
Dry mouth (n)	10	6	0.288

Values are mean (SD) or number (proportion). *Significant difference between the two groups (p < 0.05).

There were 2 cases of hypertension and 1 case of tachycardia in the control group. One case of bradycardia was observed in the study group. HR dropped to 45 beats/min (bpm) at minimum and was back to normal after treatment of atropine. No patient suffered respiratory depression and hypoxemia. Ten cases in the control group and 6 cases in the study group experienced dry mouth, although the difference between groups was not significant (p = 0.288).

## Discussion

This prospective randomized controlled multicenter study showed that the analgesic regimen including a dose of dexmedetomidine loaded immediately after delivery and postoperative sufentanil combined with dexmedetomidine for intravenous PCA could significantly relieve postoperative pain, reduce postoperative sufentanil consumption and the requirement of rescue analgesia, increase the analgesic satisfaction of the parturients, shorten the time of postoperative passage of flatus, and reduce the incidence of PONV compared to postoperative intravenous PCA using sufentanil alone without the dexmedetomidine loading dose. Furthermore, no clinically significant side effects were reported in the dexmedetomidine group.

The administration of dexmedetomidine combined with opioids in the non-pregnant population has been described in several literatures. A study revealed that a single loading dose of dexmedetomidine (1 ug/kg) 10 min before induction of general anesthesia reduced morphine consumption compared with placebo in patients undergoing abdominal surgery^11^. However, dexmedetomidine was not involved in morphine PCA after surgery in this study. The addition of dexmedetomidine to intravenous morphine PCA (5 μg dexmedetomidine per 1 mg morphine dose) improved postoperative analgesia and showed morphine-sparing effect without over sedation after abdominal total hysterectomy^12^. However an intraoperative preloading dose of dexmedetomidine was absent in this study. A study focusing on the prevention of PONV also demonstrated dexmedetomidine added to a fentany-based PCA reduced the requirement of analgesics in the early postoperative period in patients undergoing lumbar spinal fusion. But the difference of pain intensity was not the primary hypotheses in this study^13^.

Patients experience moderate to severe pain after cesarean delivery. Effective postoperative pain control can positively impact on ambulation, breastfeeding, and even maternal bonding after cesarean delivery^14^. Our previous study showed that only a loading dose of dexmedetomidine after delivery without adding dexmedetomidine into PCA did not provide extra analgesia. While, the addition of dexmedetomidine to sufentanil PCA following a loading dose of dexmedetomidine after delivery reduced the pain scores, reduced actual PCA bolus, and improved the overall satisfaction degree of mothers on analgesia within 24 h after surgery. These results indicated that sufentanil combined with dexmedetomidine enhanced the analgesic effects and provided a superior analgesic experience for the parturients.

Dexmedetomidine is a highly selective α2 receptor agonist. Due to its sedative, hypnotic, analgesic, and anti-sympathetic effects, it is very suitable for use as one component in multimodal analgesia^4,15^. Different from midazolam and propofol which sedative effects are mediated by γ- aminobutyric acid (GABA) mimetic system in the cerebral cortex, dexmedetomidine acts on the subcortical system to produce sedation aroused by language stimuli. Therefore, dexmedetomidine has been noted to provide sedation without respiratory depression^16^. These properties could be potentially beneficial to the intraoperative and postoperative safety in cesarean delivery. The sedation induced by dexmedetomidine is similar to natural sleep which may also contribute to the increase of analgesic satisfaction degree in the dexmedetomidine group.

In light of the results from the previous study^10^, the current study design was optimized with removing one treatment arm, a loading dose of dexmedetomidine after delivery and postoperative intravenous PCA without dexmedetomidine because this analgesic regimen did not enhance the analgesic effects, nor spare sufentanil. This study was focused more on clinical practice, therefore, some measures in our previous study such as pain threshold, pain tolerance threshold and plasma cortisol levels were not included in this study. Comparing with our previous studiy^10^, the administration of the PCA drugs was not based on the body weight of the individuals in this study. Instead, the PCA was programmed to deliver a 2 ml bolus on-demand with a background infusion rate of 1 ml.h^−1^ because the patients were encouraged to self-administer their own PCA medications based on their individual pain levels. Therefore, the administration of drugs based on body weight was not necessary. Additionally, this optimized protocol was more practical in clinical practice and more convenient to execute by investigators in multiple centers without bias.

Studies have shown that paracetamol was not better than placebo at relieving pain from uterine cramping/involution^17^. From the perspective of study design, if other adjuvants (acetaminophen, non-steroidal anti-inflamitory drugs) were included in the analgesic regimen, different plasma concentration of the adjuvants resulted from the different PCA consumption between the groups could have misled or hidden the effect of dexmedetomidine. These potential confounding factors should be controlled as far as possible. Therefore, acetaminophen and nonsteroidal anti-inflammatory drugs (NSAIDS) which are routinely used in clinical context were avoided in this study.

The present study didn’t show significant difference in the sedative degree between the two groups, which was consistent with the result of our previous study. Although dexmedetomidine had sedative functions, the OAA/S sedation scores in this study and the Ramsay sedation scores (RSS) used in our previous study did not show the presence of excessive sedation, which indicated that the dose of dexmedetomidine used in our study protocol did not produce a level of sedation with clinical significance but might increase the analgesic satisfaction of the patients.

Although no differences were detected in other hemodynamic indicators except HR, the HRs in the dexmedetomidine group were lower than those in the control group at 5, 10, and 30 mins after the study drugs administration, indicating that dexmedetomidine slowed down the HR. The meta-analysis by Blaudszun *et al*. showed that dexmedetomidine had a risk of inducing postoperative bradycardia^18^. However, this meta-analysis targeted the non-pregnant population, and the definition of bradycardia in each study was different. Therefore, the risk of the induction of bradycardia in healthy pregnant women was uncertain. Only one case with mild bradycardia was reported in this multicenter study, which was rapidly corrected after the administration of atropine.

This study showed that the addition of dexmedetomidine into sufentanil PCA reduced the incidence of PONV. Previously, our single-center study did not show this potential benefit of dexmedetomidine, which might be associated with the increase in the sample size and the multicenter design in this study. This weak anti-nausea function could be explained by the direct anti-nausea and vomiting functions of α2 receptor agonists. Although the biological theory underlying this function is not clear, PONV might be associated with the high postoperative concentration of catecholamine^19^. Additionally, the anti-sympathetic characteristic of dexmedetomidine might reduce PONV. Furthermore, dexmedetomidine reduced postoperative sufentanil consumption. Opioid drugs are risk factors for PONV. Therefore, the reduced consumption may have indirectly reduced the incidence of PONV.

Few large sample-size studies have examined postoperative obstetric analgesia with dexmedetomidine. Although *in vitro* studies showed that a low concentration of dexmedetomidine (1 × 10^−9^ g.ml^−1^) could induce uterine contractions^20^, the clinical study conducted by El-Tahan *et al*. showed that dexmedetomidine increased uterine tension in cesarean delivery under general anesthesia and reduced the use of oxytocin^7^. However, in our study, there was no significant difference between the two groups in the amount of lochia discharge within the first 3 h after surgery. This discrepancy might be related to the large individual variations in the amount of lochia with large SD. With the exception of the postoperative 8 h time point, dexmedetomidine did not reduce uterine contraction pain at any of the time points, possibly due to the collective effect of the analgesic function of dexmedetomidine and the uterine contraction function. The time of postoperative passage of flatus in the dexmedetomidine group was shortened, suggesting that the anti-sympathetic function of dexmedetomidine and the reduction of sufentanil consumption might facilitate the recovery of the intestinal functions of patients after surgery.

This study has some limitations. Firstly, the sample size was calculated based on the primary outcome. Thus, this study was not sufficiently powered to address the differences in medical events with a very low incidence between the groups, such as hypotension and bradycardia. The definition of bradycardia in this study was a HR <50 bpm, which was higher than that in previous study^21,22^ and might have overestimated the incidence of bradycardia. Therefore, we speculated dexmedetomidine should not cause concern about bradycardia in healthy parturients. The population selected in this study contained healthy parturients without complicated pregnancies. Therefore, the analgesic program in this study was not applicable for parturients with complicated pregnancies and dexmedetomidine contraindications, such as bradycardia prior to surgery, atrioventricular blocks, and hypovolemia. Secondly, this study did not measure the dexmedetomidine concentration in breast milk. Thus the drug exposure level of the newborns remains unclear. This unclear exposure level requires further studies focused on the risk of exposure to dexmedetomidine through breast milk in newborns. However the onset of lactation in this study was 34.3 h in the control group and 32.6 h in the dexmedetomidine group. The elimination half-life of dexmedetomidine was 2 h. The package insert of dexmedetomidine indicates that approximately 85% of the radioactive substances were excreted in the urine within 24 h after the radioactive labeling dexmedetomidine infusion. Therefore, we hypothesized that the plasma dexmedetomidine level was already low after the start of lactation in the majority of the parturients. However, there were large individual variations in the onset of lactation, and we could not exclude that there was still a certain concentration of dexmedetomidine in the circulation of individual parturients after the initiation of breastfeeding. The pharmacological characteristics of dexmedetomidine such as high protein-binding rate (94%), slight acid compared with plasma and large molecular size, ensure that it can’t easily enter lactated breast milk. As dexmedetomidine has lower oral bioavailability (16%), we speculated that its effect is negligible even though a small amount of dexmedetomidine is ingested by neonate via breast milk.

In summary, the addition of dexmedetomidine to sufentanil intravenous PCA following a loading dose after delivery could significantly enhance the analgesic effects, reduce sufentanil consumption, and increase the analgesic satisfaction of parturients compared to the use of sufentanil alone for intravenous PCA. Additionally, this analgesic program was beneficial for the recovery of the intestinal functions and had potential benefits for the incidence reduction of PONV.

## Materials and Methods

This prospective, randomized, controlled, multicenter study was approved by the central Ethics Committee of the Obstetrics and Gynecology Hospital of Fudan University and the other 8 medical centers accepted the approval of central Ethics committee. This study was conducted in compliance with China Good Clinical Practice (GCP) and the tenets of the Declaration of Helsinki. This study was registered in ClinicalTrials.gov by Yuyan Nie on April 13 (rd), 2016 (registration number: NCT02741219, URL: https://clinicaltrials.gov/ct2/show/NCT02741219). Written informed consent was obtained from all parturients. Nulliparous parturients who had full term singletons, scheduled for cesarean delivery under combined spinal-epidural anesthesia and had request for postoperative intravenous anesthesia were enrolled into this study. The inclusion criteria were American Society of Anesthesiologists (ASA) I or II, age between 18–45 years, able to fully communicate and correctly use the PCA device. The exclusion criteria were as follows: (1) known allergy to dexmedetomidine or the other drugs used in this study; (2) long term use of narcotic analgesics, sedatives, or non-steroidal anti-inflammatory drugs; (3) a history of neuromuscular diseases, endocrine system diseases, allergic diseases, or mental illness; (4) a history of middle or lower abdominal surgery; (5) preoperative HR <50 bpm, SBP <100 mmHg, and abnormal cardiac conduction or rhythm; (6) failed CSEA anesthesia or required epidural drugs to achieve satisfactory anesthetic effects; (7) a second surgery was required during the observation period; (8) participation in other drugs clinical studies during the past 3 months; (9) BMI >30 kg.m^−2^; and (10) suspected difficult airways (including anatomical abnormalities and a modified Mallampati score >3). Parturients who did not meet the inclusion criteria or met the exclusion criteria after enrollment were excluded from this study. Moreover, women who experienced severe adverse reactions or anesthesia accidents were also eliminated.

Randomization was performed separately in nine participating centers. Spinal blocks were performed with the fixed dose. The type of the local anesthetic was left to the discretion of the research anesthesiologist with no restriction regarding their choice (ropivacaine or bupivacaine). But patients in the same center were used the same local anesthetic. According to a computer-generated random sequence which was created by Microsoft Office Excel, patients were allocated to one of two groups. Treatment allocation was kept concealed in numbered and opaque envelopes which were opened consecutively after the parturients had entered the study. Prior to anesthesia, the parturients were visited to introduce this study and to obtain signed informed consent. After the parturients entered the operation room, the random envelopes were opened. A nurse anesthetist prepared the experimental drugs and the PCA pumps based on the treatment allocation. A research anesthesiologist blinded to the group allocation was involved in applying the experimental drugs, postoperative patient care and data collection. The parturients were also unaware of their grouping conditions.

### Anesthesia methods

During the preoperative visit, patients were instructed on the proper use of VAS for assessing pain and the PCA pumps. Though restriction in the brands of analgesic pumps was not required, each PCA device was able to collect all data in our protocol. No premedication was administered before the operation. Noninvasive blood pressure (NBP), electrocardiogram (ECG), HR, and SPO_2_ were routinely monitored. After entering the operation room, the parturients assumed a supine position on the operation bed, and NBP was measured after 5 minutes and 10 minutes. The mean value of these two measurements was used as the baseline blood pressure prior to anesthesia. Hydroxyethyl starch (6%) was rapidly infused at 20 ml/min before anesthesia puncture. All participants received CSEA at L3-4 or L2-3 space using the needle-through-needle technique. After subarachnoid injection with 15 mg ropivacaine or 10 mg bupivacaine (the two groups were consistent within the same center) was accomplished, an epidural catheter was rapidly inserted cephalad 3–4 cm. Then patients were positioned supine with left uterine displacement until delivery. The surgery started when the T6 sensory block was achieved. The epidural catheter was used only when the block was not sufficient or the surgical time needed to be extended. Once the surgery began, neuraxial local anesthetics and any intravenous analgesic or sedative drugs were no longer used. Oxygen was administered by face mask at 5 L/min.

If hypotension occurred (SBP <90 mmHg or the reduction greater than 20% of the basal SBP), 50 µg of phenylephrine was administered intravenously, and the fluid infusion was speeded up. When the SBP was higher than 180 mmHg, the infusion speed was lowered and 25 mg of urapidil or 0.1 mg of nicardipine was administered intravenously. If the HR was lower than 50 bpm, 0.2 mg of atropine was intravenously injected. If the HR was higher than 110 bpm, 10 mg of esmolol was used intravenously. The above drugs used for the maintenance of hemodynamics could be repeatedly administered if necessary. If the parturients shivered during the surgery, 50 mg of tramadol was intravenously infused. Prior to abdominal closure, 4 mg of ondansetron was routinely intravenously use to prevent PONV.

The parturients were sent to post-anesthesia care units (PACU) after surgery. Vital signs including SBP/DBP, oxygen saturation, ECG, and HR were monitored continuously and recorded with 15 minutes interval in PACU. The sensory level was assessed with pinprick by the research anesthesiologist blinded to the group allocation every 15 minutes. When vital signs were stable and the sensory block was not higher than T8, the intravenous PCA pumps were connected in PACU, and then the parturients were sent back to their ward. PCA pumps were discontinued after 24 h for all subjects. If the parturients still complained about pain during PCA, rescue analgesia was administered by intramuscular injection of 100 mg tramadol; if necessary, the injection could be repeated.

### Allocation of subjects

(1) Control group: Intravenous infusion of 20 ml of normal saline (NS) was performed 10 min after fetal delivery; the infusion lasted 10 min. The sensory block level decreased below T8 after surgery, the intravenous PCA pump was connected for 100 μg sufentanil + NS in a total volume of 100 ml; the background infusion was 1 ml.h^−1^ (sufentanil 1 μg.h^−1^), the bolus dose was 2 ml (sufentanil 2 μg), and the lock time was 8 min. (2) Study group: A loading dose 0.5 µg/kg of dexmedetomidine was administered over 10 min after delivery (dexmedetomidine was prepared as a 20 ml solution using NS). When the sensory block level lower than T8, the intravenous PCA pump was connected for 100 μg of sufentanil +300 µg of dexmedetomidine + NS in a total volume of 100 ml. The PCA background infusion was 1 ml.h^−1^ (sufentanil 1 μg.h^−1^, dexmedetomidine 3 μg.h^−1^), the bolus dose was 2 ml (sufentanil 2 μg, dexmedetomidine 6 μg), and the lock time was 8 min.

### Main outcome measure

The total amount of postoperative sufentanil consumption within 24 h after surgery was the main outcome, which could be calculated according to the record of PCA device.

### Secondary outcome measures

Pain intensity was evaluated with VAS score, in which 0 cm means no pain and 10 cm represents the worst imaginable pain. Characterized as a type of cramping pain, uterine contraction pain was defined as intermittent pain in the lower abdomen, and occurs due to the constant and rhythmic contractile activity of the uterus. Postoperative VAS scores (at rest, on movement and uterine contraction pain) were recorded at 0, 4, 8, and 24 h after surgery. The total number of times that the PCA button was pressed (total bolus), and the number of actual boluses delivered were measured based on the data recorded by the PCA device.

The analgesic satisfaction degree of parturients was evaluated at 24 h after surgery and was generally divided into 4 grades (dissatisfied, acceptable, satisfied, and very satisfied). Satisfaction degree for analgesia was compared between two groups. The number of rescue analgesia within 24 h after surgery was also recorded.

The degree of sedation were measured with the modified Observer’s Assessment of Alertness/Sedation Scale (OAA/S) as follows: 5 points indicated a sensitive response to the name spoken in a normal tone and complete alertness; 4 points indicated a lethargic response to the name spoken in a normal tone and a slower speaking speed; 3 points indicated a response only after the name was called loudly and/or repeatedly; 2 points indicated a response only after mild prodding or shaking; 1 point indicated no response to mild prodding or shaking; and 0 points indicated no responses to noxious stimulation. The sedation levels of the parturients were scored postoperatively at 0, 4, 8, and 24 h.

Additionally, the incidence of PONV within the first 24 h after surgery and the use of antiemetic were also recorded.

The onset of lactation was defined as below: Parturients perceived strong symptoms of breast tension, heat and pain and had the objective signs including formation of hard milk knots, exposed or varicose veins on the surface of the breasts. Meanwhile more than 10 ml milk flowed after both breasts were massaged by the bedside nurse. If the subjective major symptoms related to milk arrival became strong, breasts massage was done at regular 2 h intervals or according to the request of mothers until lactation. We recorded the onset time of lactation as documented by the bedside nurse (minutes).

The amount of lochia within the first 3 h after surgery by weighing the sanitary napkins and the first passage of flatus were also noted.

The SBP, DBP, MAP, HR, and SpO2 were recorded before surgery and at 0 min, 5 min, 10 min and 30 min after drug administration during surgery and at 8 h, 24 h postoperatively.

ECG and SpO_2_ were monitored continuously during postoperative analgesia. Oxygen was administered at 4 L/min by nasal tube when maternal SpO_2_ was lower than 95% in room air. When the SpO_2_ remained <95% after oxygen administration, the background infusion of the PCA pump was reduced by half. When SpO_2_ was less than 90% in the case of oxygen inhalation, the analgesic pump was temporarily stopped, and assisted ventilation was performed.

Adverse events that occurred between the administration of study drugs and 24 h after surgery were recorded including hypotension (SBP <90 mmHg or a more than 20% reduction compared to the SBP prior to the infusion of the experimental drugs), hypertension (SBP >180 mmHg or a more than 20% increase of the SBP prior to the administration of the study drugs), bradycardia (HR <50 bpm), tachycardia (HR >110 bpm), respiratory depression (the respiratory rate was lower than 10 times/min and lasted more than 10 min), and hypoxemia (SpO_2_ <95% during oxygen inhalation).

Postoperative hemodynamic indicators, postpartum lactation, the lochia, PONV were recorded by the ward nurse who took charge of the patient. The other data was collected by the same research anesthesiologist who was designated for this study in each center.

### Statistical analysis

The primary outcomes were the sufentanil consumption after surgery. Secondary outcomes included in pain scores, the requirement of rescue analgesia, sedation scores, analgesic satisfaction, the incidence of PONV and time to first passage of flatus within 24 h after surgery and hemodynamic changes after study drug administration.

Measurement data are shown as means ± standard deviations (M ± SD) or mean difference with 95% confidence interval [MD, 95% CI]. Categorical data are presented as cases and frequency. The Kolmogorov-Smirnov test was performed to test whether the data conformed to a normal distribution. Comparisons of measurement data that conformed to the normal distribution were performed using the unpaired t test. Hemodynamic data included SBP, DBP, MAP and HR before surgery, at 0 min, 5 min, 10 min, 30 min after loading drug administration during surgery and at 8 h, 24 h after surgery. Intergroup differences in repeated measures such as VAS pain scores and hemodynamic data were assessed by two-way (time and group) repeated measures analysis of variance (ANOVA) and Bonferroni post hoc test was used to adjust for multiple comparisons. The longitudinal analysis with the linear mixed models (LMMs) procedure was also used to compare the overall changes over time in VAS pain scores between the groups. Categorical data including the incidence of PONV and the OAA/S sedation scores were examined using the χ2 test or Fisher’s exact test. The statistical analyses were performed using SPSS (Version 13.0, SPSS Inc., Chicago, IL, USA) software. P values <0.05 were considered statistically significant.

The sufentanil consumption was the main outcome in this study. The sample size was calculated by the difference of sufentanil consumption. Assuming that the true difference between groups is 10.6 ug and common standard deviation for each group is 21.7 ug^10^, a total of 168 patients with 1:1 randomization ratio were required to achieve 80% power at a 2-sided alpha level of 5% for detection of difference between the two groups. We added 20% more patients to account for drop-outs during the study.

### Nine Participanting Medical Centers

The Obstetrics and Gynecology Hospital of Fudan University, Tongji Medical College Huazhong University of Science & Technology, General Hospital of Guangzhou Military Command of PLA, Renji Hospital Shanghai Jiaotong University School of Medicine, Tianjin Medical University General Hospital, The Affiliated Hospital of Qingdao University, Nanjing Maternity and Child Care Hospital, Sun Yat-Sen Memorial Hospital of Sun Yat-Sen University, Guangzhou Women And Children’s Medical Center.
